# Testosterone Deficiency in Sickle Cell Disease: Recognition and Remediation

**DOI:** 10.3389/fendo.2022.892184

**Published:** 2022-05-03

**Authors:** Biljana Musicki, Arthur L. Burnett

**Affiliations:** Department of Urology, The James Buchanan Brady Urological Institute, The Johns Hopkins School of Medicine, Baltimore, MD, United States

**Keywords:** nitric oxide, testosterone replacement, TSPO, PDE5, erectile dysfunction, infertility, oxidative stress

## Abstract

Hypogonadism is common in men with sickle cell disease (SCD) with prevalence rates as high as 25%. Testicular failure (primary hypogonadism) is established as the principal cause for this hormonal abnormality, although secondary hypogonadism and compensated hypogonadism have also been observed. The underlying mechanism for primary hypogonadism was elucidated in a mouse model of SCD, and involves increased NADPH oxidase-derived oxidative stress in the testis, which reduces protein expression of a steroidogenic acute regulatory protein and cholesterol transport to the mitochondria in Leydig cells. In all men including those with SCD, hypogonadism affects physical growth and development, cognition and mental health, sexual function, as well as fertility. However, it is not understood whether declines in physical, psychological, and social domains of health in SCD patients are related to low testosterone, or are consequences of other abnormalities of SCD. Priapism is one of only a few complications of SCD that has been studied in the context of hypogonadism. In this pathologic condition of prolonged penile erection in the absence of sexual excitement or stimulation, hypogonadism exacerbates already impaired endothelial nitric oxide synthase/cGMP/phosphodiesterase-5 molecular signaling in the penis. While exogenous testosterone alleviates priapism, it disadvantageously decreases intratesticular testosterone production. In contrast to treatment with exogenous testosterone, a novel approach is to target the mechanisms of testosterone deficiency in the SCD testis to drive endogenous testosterone production, which potentially decreases further oxidative stress and damage in the testis, and preserves sperm quality. Stimulation of translocator protein within the transduceosome of the testis of SCD mice reverses both hypogonadism and priapism, without affecting intratesticular testosterone production and consequently fertility. Ongoing research is needed to define and develop therapies that restore endogenous testosterone production in a physiologic, mechanism-specific fashion without affecting fertility in SCD men.

## 1 Introduction

Sickle cell disease (SCD) is the most common hereditary hematologic disorder in the United States, which affects an estimated 100,000 Americans, mostly African-Americans, and millions of people globally (1). Patients with SCD experience acute complications, such as painful vaso-occlusive episodes, and chronic multi-organ damage, which heighten their risks for morbidity and mortality (2). SCD was long considered to be a disease of children and young adults because of its devastating natural progression. Due mostly to universal newborn screening and early therapeutic intervention, life expectancy in patients with SCD has steadily improved over the last 30 years, and recent studies have estimated the median survival for patients with SCD at 60 years (3). Extended survival outcomes have, however, led to an increase in long-term complications of this disease.

SCD is associated with hypogonadism (total testosterone levels below 300 ng/dl), which develops in up to 25% of men with this disease (4). This rate contrasts with the 6-12% prevalence rate of symptomatic hypogonadism in otherwise healthy middle aged and older men, who manifest an age-related decline in testosterone production (5). The impact of testosterone deficiency in the SCD male population is evident, based on its symptomatic effects, e.g., impaired physical and sexual maturation, reduced libido, erectile dysfunction, decreased physical strength, fatiguability, mood changes, and infertility (6, 7). Attempts to address this problem are, however, hampered by limited understanding of the mechanism of hypogonadism in SCD.

This review focuses on the mechanism of testosterone deficiency in SCD, the impact of hypogonadism on health- and reproduction-related issues in SCD males, and novel strategies to drive endogenous testosterone biosynthesis. These strategies may translate into clinical therapeutic opportunities for preserving sexual function and fertility, and possibly other conditions, adversely affected by hypogonadism in SCD.

## 2 Sickle Cell Disease

SCD is caused by a single point mutation in the β-globin gene of hemoglobin, leading to the expression of abnormal sickle hemoglobin (HbS). Traditionally, the pathophysiology of SCD was thought to result exclusively from the polymerization of HbS under hypoxic conditions, causing erythrocytes to become deformed, sludge, and occlude blood vessels, along with oxidative stress, inflammation, and hemolytic anemia (8). More recent studies show that SCD is also characterized by a chronic deficiency of the endogenous vasodilator nitric oxide (NO) and vascular dysfunction (8, 9). As a consequence, SCD leads to progressive multi-organ failure resulting in pulmonary hypertension, leg ulcers, renal failure, stroke, infarct, retinopathy, neurocognitive impairment, bone loss, and priapism (2, 9, 10).

### 2.1 Hypogonadism in Sickle Cell Disease

Clinical research has documented a high frequency of testosterone deficiency in SCD, with prevalence rates as high as 25% (4). In a small number of clinical studies investigating hypogonadism in SCD, findings regarding its etiology and clinical implications have varied. Studies have reported elevated luteinizing hormone (LH) and follicle-stimulating hormone (FSH) levels in patients with SCD (primary hypogonadism; 6, 11–14). Repeated testicular infarction is observed in some men with SCD, attributed to erythrocyte sickling, obstructed blood flow, and hypoxia (15), and this course has been proposed to be a contributing factor for testicular failure (16–19). In contrast, studies report decreased LH and FSH in patients with SCD (secondary hypogonadism; 4, 20, 21). Furthermore, compensated hypogonadism (characterized by increased gonadotropins and normal testosterone levels) has also been identified in men with SCD (22). Smaller testis size in SCD men (6, 23) and reduced testis weight in SCD mice (24) is further evidence of hypogonadism related to this disease.

In recent years, progress has been made toward understanding the mechanism of testosterone deficiency in SCD, and primary hypogonadism has now been established as the principal cause for this hormonal abnormality. Oxidative/nitrosative stress is implicated in defective testosterone production by affecting the expression or enzymatic activation of several steroidogenic enzymes, or by depletion of antioxidants (25–27). In the vasculature of humans and experimental animals with SCD, reactive oxygen species (ROS)-generated enzymes NADPH oxidase (NOX) and xanthine oxidase, endothelial NO synthase (eNOS) uncoupling, autooxidation of HbS, heme iron release, and increased asymmetric dimethylarginine have been described (28, 29). Diverse stimuli associated with these redox sources include hypoxia, angiotensin II, proinflammatory cytokines, vasoconstrictors, growth factors, metabolic factors, and superoxide itself (30).

The testis of the SCD mouse exhibits upregulation of 4-hydroxy-2-nonenal (4-HNE), a major end product of lipid peroxidation, upregulation of NOX gp91phox subunit, and uncompensated expression of the antioxidant enzyme glutathione peroxidase-1, all consistent with a heightened and uncontrolled redox environment in the SCD mouse Leydig cell (31). Increased NOX-derived oxidative stress reduces protein expression of steroidogenic acute regulatory protein (StAR) (but not cholesterol side-chain cleavage enzyme) in Leydig cells of the SCD mouse testis, which initiates cholesterol transfer into mitochondria. Reduced transport of cholesterol to mitochondria of Leydig cells in the SCD testis accounts for primary hypogonadism (31).

Secondary hypogonadism appears to represent patients having more severe or progressive forms of SCD, who exhibit more frequent abnormalities of LH and FSH in comparison with patients having mild disease (20). While not completely understood, secondary hypogonadism may be the result of vasoocclusion of hypothalamic-pituitary small blood vessels, or pituitary infarction (11).

#### 2.1.1 Hypogonadism, Reproductive Issues, and Health-Related Quality of Life in SCD

Testosterone plays a critical role in muscle physiology, body development, bone density, sexual function, fertility, as well as social, emotional, and neurocognitive functioning in males (32). Patients with SCD exhibit reduced height and weight, decreased physical strength, and delayed sexual maturation (23). Low levels of testosterone have been associated with very low bone mass density in SCD patients compared with those having normal bone mass density (33). Psychological distress, such as mood changes, increased anxiety, extreme fatigue, social withdrawal, and depression, and neurocognitive impairment, such as impaired executive function, attention, and processing speed, are well recognized complications of SCD (34–36). However, it is not understood whether declines in physical, psychological, and social domains of health in SCD patients are related to low testosterone levels or are consequences of other abnormalities of SCD. Future studies are warranted to evaluate this possible consequence of hypogonadism in SCD.

Although poorly studied in SCD, male infertility is recognized to be a common complication of this disease (23, 37–39). Impaired male fertility in SCD is due to multiple causes, including hypogonadism, gonadal failure and sperm abnormalities (such as oligospermia, reduced sperm motility and density, and abnormal sperm morphology), decreased ejaculate volume, and delayed or impaired sexual development. Prevalence rate of at least one abnormal sperm parameter in male patients with SCD is 91% (40). Erectile dysfunction, largely as a result of penile damage from recurrent or prolonged priapism, further contributes to reduced fertility in SCD men (23).

#### 2.1.2 Hypogonadism and SCD-Related Priapism

Priapism is a pathologic condition of prolonged penile erection in the absence of sexual excitement or stimulation (41). Ischemic priapism, which features little or absent intracorporal blood flow resulting in painful erections, is prevalent in men with SCD, occurring in as many as 48% of men, with a mean age of onset of 15 years (42, 43). Repeated episodes of priapism may lead to irreversible damage to erectile tissue and permanent erectile dysfunction (42, 44, 45) and cause psychological distress, impaired sexual relationships, and reduced quality and function of life (46). The prevalence rate of erectile dysfunction associated with recurrent ischemic priapism in SCD patients is as high as 47.5% (47).

The historical premise is that androgens are causative in the pathophysiology of priapism. However, this notion is now challenged. Reports of no increase in priapism in testosterone deficient men administered testosterone gel at eugonadal levels (48), as well as reduced priapism occurrences in testosterone deficient men with SCD receiving long-acting testosterone undecanoate injections (49) oppose earlier conceptions that testosterone therapies cause priapism. It is now established that physiologic testosterone administration does not cause priapism and, in contrast, this intervention promotes molecular mechanisms that favor normal erection responses. In fact, priapism in SCD is associated with decreased testosterone levels. A potential role for testosterone in correcting priapism acknowledges that androgens contribute to physiologic erectile tissue responses. Testosterone and dihydrotestosterone promote physiologic relaxation of penile arteries and cavernous tissue, and androgen deficiency decreases the expression and enzymatic activities of eNOS, neuronal NOS, and phosphodiesterase type 5 (PDE5) in the penis, the main players in penile erection (50).

The mechanisms by which testosterone deficiency contributes to priapism has recently been elucidated. In a mouse model of SCD, characterized by both primary hypogonadism and priapism (51), testosterone replacement at eugonadal levels corrects priapism. At the molecular level, normalized testosterone levels reverse downregulated eNOS activity *via* a nongenomic mechanism by normalizing downregulated P-Akt (Ser-473) and P-eNOS (Ser-1177) protein expressions in the penis (51). Increased NO reverses downregulated protein expression and activity of PDE5, the enzyme which degrades cGMP in the penis (52–56). Testosterone’s effect on PDE5 protein expression is believed to be mediated by increased NO-induced accumulation of cGMP, which binds to cGMP response sequences in the PDE5 promoter (57). Testosterone’s effect on PDE5 catalytic activity is due to phosphorylation of PDE5 on Ser-92 by cGMP-mediated activation of protein kinase G, which stimulates binding of cGMP to the regulatory domain of PDE5 (58). Upregulated PDE5 protein expression and activity in the penis restores the mechanism for cGMP degradation, thereby preventing excessive accumulation of this nucleotide upon neurostimulation. By controlling the amount of cGMP, which causes relaxation of smooth muscles in the penis and penile erection, priapic activity is lessened (51). This proof-of-principle study supports testosterone deficiency as a cause for SCD-associated priapism by exacerbating already impaired NO molecular signaling in the penis.

In contrast to its physiologic doses, testosterone at supraphysiologic doses decreases NO production from eNOS and increases oxidative stress in endothelial cells (59–61). This may partially explain findings described in several case reports in men that, at excessive dosing, testosterone may trigger priapism rather than reduce it (62–64).

Priapism is one of very few complications of SCD that has been studied in the context of hypogonadism. It is interesting to observe that low testosterone exhibits opposing erection phenomena in the general population of men vs men with SCD: while low testosterone may contribute to decreased erection in the general population having cardiovascular or metabolic factors affecting erectile tissue function, it results in uncontrolled erection in the SCD population, which has a severely disturbed PDE5 regulatory pathway in the penis. However, it is noted that achieving physiologic “eugonadal” effects in the penis is healthful in both populations.

## 3 Testosterone Replacement Strategies

Traditional approaches for managing testosterone deficiency in general have largely centered on exogenous administration of testosterone. Testosterone therapies and their relative usages are: transdermal testosterone gel therapy (70%), testosterone injections (17%), transdermal testosterone patches (10%), and other forms of testosterone therapy, such as an oral formulation (3%) (65, 66). However, limitations exist with these current therapies. Adverse side effects are commonly described in association with exogenous testosterone administration, including supraphysiologic levels of testosterone, local irritation with applications, gynecomastia, erythrocystosis, hepatotoxicity, and sleep apnea (67). Adverse prostate health risks of benign prostate enlargement and prostate cancer as well as cardiovascular risks (i.e., edema, heart attack, stroke) have also been contended to be potential risks of testosterone therapy (68). Impaired sperm production and infertility are also documented risks of exogenous testosterone therapies, by virtue of feedback inhibition of central gonadotropin release. Such therapies suppress LH, which in turn suppress Leydig cell-stimulated testosterone production, resulting in reduced intratesticular testosterone concentrations needed for spermatogenesis (67, 69). Because of the contraceptive effect exerted by exogenous testosterone preparations, many young men with hypogonadism desiring to retain reproductive function are precluded from pursuing exogenous testosterone therapies as a therapeutic option.

Alternatives to exogenous testosterone treatment have been explored, with the main objective to drive endogenous testosterone production and in turn preserve fertility. Current options include selective estrogen receptor modulators (SERMs), aromatase inhibitors, and human chorionic gonadotropin (hCG) (70). Both SERMs (e.g., clomiphene citrate and tamoxifen citrate), which serve as estrogen receptor antagonists, and aromatase inhibitors (e.g., letrozole, anastrozole, and testolactone), which block the conversion of testosterone to estradiol, result in decreased estrogen feedback to the hypothalamus thereby effecting a natural increase in gonadotropin release (70). Their efficacy in increasing testosterone production is limited in men with normal or elevated LH levels who manifest a testosterone production defect at the testicular level. hCG, operating as an LH analogue, serves to stimulate Leydig cell production of testosterone. Its efficacy is limited in men whose Leydig cells are not functionally responsive to LH because of decreased receptor function or capacity for testosterone production (65, 71).

These reports indicate that currently available testosterone therapeutic options aiming to enhance endogenous testosterone production fall short in addressing testosterone deficiency associated with testicular failure. This shortcoming is relevant generally and for hypogonadal males with SCD. Specifically in males with SCD, exogenous testosterone would further affect fertility by decreasing intratesticular testosterone production needed for spermatogenesis.

## 4 Endogenous Mechanism-Specific Molecular Targets for Testosterone Production

Targeting mechanism-specific endogenous sources of testosterone production in the SCD testis to produce eugonadal levels of the hormone directly addresses primary hypogonadism. As transfer of cholesterol from the outer to the inner mitochondrial membrane of Leydig cells in the testis is the principal site of regulation of steroid hormone biosynthesis, and is impaired in SCD, targets for stimulating testosterone production may involve transduceosome protein components. The transduceosome is an ensemble of mitochondrial and cytosolic proteins responsible for cholesterol translocation from intracellular stores to the inner mitochondrial membrane (72). Translocator protein (TSPO) is a high-affinity drug- and cholesterol-binding mitochondrial protein, and its protein expression is decreased in the testis of SCD mice (73, 74). The TSPO-dependent import of StAR into mitochondria and the association of TSPO with the outer/inner mitochondrial membrane contact sites drives intramitochondrial cholesterol transfer and subsequent steroid formation (73). Previous studies have shown that TSPO drug ligands activate steroid production by MA-10 mouse Leydig tumor cells and by mitochondria isolated from other steroidogenic cells (75–77). Furthermore, pharmacologic stimulation of TSPO stimulates testosterone production, both *in vitro* by Leydig cells isolated from aged rats and *in vivo* in aged rats, without reducing intratesticular testosterone concentrations or sperm number (78, 79). These studies oppose several previous reports which questioned the role and extent of involvement of TSPO in mitochondrial cholesterol import and steroidogenesis (80, 81).

A recent study in a SCD mouse model demonstrated that pharmacologic stimulation of TSPO corrects priapism. Treatment of SCD mice with TSPO-selective drug ligand N,N-dihexyl-2-(4-fluorophenyl) indole-3-acetamide (FGIN-1-27) produces eugonadal levels of testosterone. Normalized testosterone levels corrects priapism without decreasing intratesticular testosterone production (74). At the molecular level, TSPO ligand, by normalizing testosterone levels, restores PDE5 activity and decreases NOX-mediated increase in oxidative stress in the penis. Conceivably, this effect of testosterone pertains to recovered control of NO/cGMP responsiveness associated with restored PDE5 function. The mechanism underlying testosterone’s inhibitory effect on NOX expression and activity is not known, but may be indirect through the improvement of endothelial function. In human endothelial cells and mouse aorta, NO S-nitrosylates and inhibits p47phox subunit of NOX, inhibits protein expression of gp91phox and p47phox subunits of NOX, and inhibits superoxide production (82–84). These findings suggest that targeting endogenous testosterone production in the SCD testis by pharmacologic activation of protein components involved in cholesterol transport could be a novel, targetable pathway to correct primary hypogonadism and ameliorate testosterone deficiency-associated health conditions without affecting fertility.

**Figure 1:**
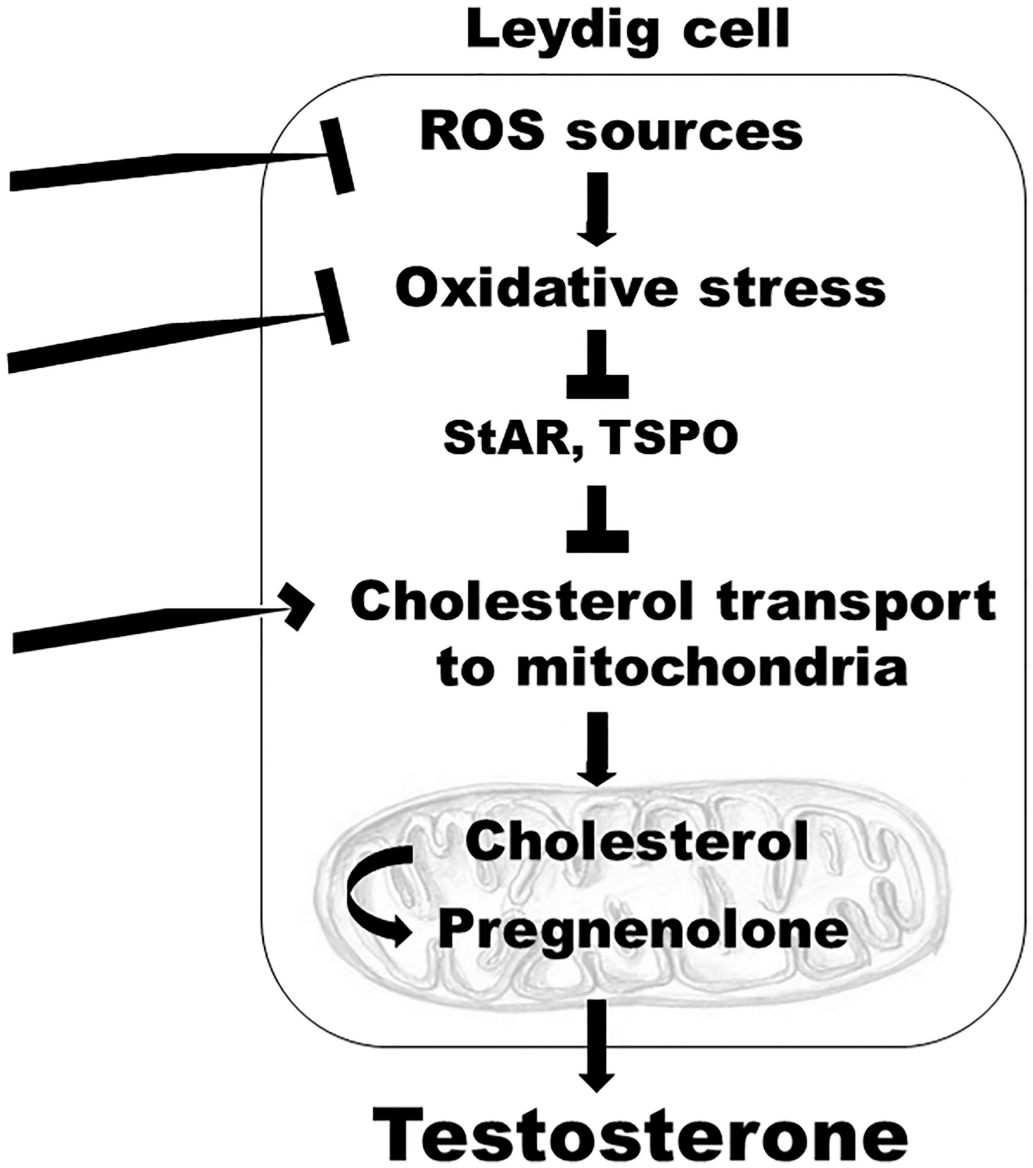
A model depicting mechanism-specific endogenous targets within the Leydig cells of the testis which can be modulated to reverse hypogonadism in SCD. Targets include: inhibition of enzymatic sources of oxidative stress, such as NOX; inhibition of increased oxidative stress, which decreases protein synthesis of enzymes involved in cholesterol transport to the mitochondria, such as StAR and TSPO; stimulation of cytosolic or outer mitochondrial membrane protein components involved in cholesterol transport from intracellular stores to the inner mitochondrial membrane; ROS, reactive oxygen species; StAR, steroidogenic acute regulatory protein; TSPO, translocator protein.

While not examined, it is plausible that, in addition to TSPO, other cytosolic or outer mitochondrial membrane protein components involved in cholesterol transport from intracellular stores to the inner mitochondrial membrane (such as voltage dependent anion channel 1, negative protein adaptor 14-3-3ϵ, or AAA domain-containing protein 3A) (72), may be targeted in the SCD testis to increase endogenous testosterone production. Because pharmacologic activation of TSPO is independent of LH, it is conceivable that this approach may treat secondary hypogonadism, or mixed primary and secondary hypogonadism, as well. Other possible mechanism-based targets in the SCD testis include increased oxidative stress, or enzymatic sources of oxidative stress (such as NOX), which are enhanced in SCD-associated primary hypogonadism (**Figure 1**).

Of note, L-glutamine, one of the 3 recently FDA-approved treatments for SCD (L-glutamine, crizanlizumab, and voxelotor), increases glutathione-dependent anti-oxidation in the testis and testosterone levels, at least in sleep-deprived rats (85), while alleviating primary hypogonadism and protecting erythrocytes against oxidative damage.

## 5 Discussion

SCD affects millions of people throughout the world, mostly of African ancestry, and is recognized by the World Health Organization and United Nations as a global health issue. In the United States, health outcomes for people with SCD have improved in the past few decades. Despite medical advances, life expectancy for individuals with SCD in the United States remains 20 to 30 years lower than that of the average American. It has been recognized that research and treatment efforts for SCD lag behind that of other chronic genetic illnesses, such as hemophilia and cystic fibrosis, requiring legislative attention (86, 87). In correlation, less FDA-approved therapies are currently available for SCD. The Sickle Cell Disease Comprehensive Care Act, signed into law in December 2018, represents a commitment by the government to continue research towards increasing the understanding of prevalence, distribution, outcomes, and therapies associated with SCD.

Amidst health care disparities among ethnic populations in the United States, limited knowledge and action surround hypogonadism in SCD, in spite of its long-term and costly health problems. While many studies have evaluated the mechanism and health-related issues of hypogonadism in the general adolescent population, very few studies have focused on hypogonadism in the SCD population. For example, although an estimated 1 in 4 SCD patients exhibits low testosterone levels, no studies have assessed the testosterone-dependent health-related quality of life profiles of SCD patients.

Despite inequity in federal and foundation research funding, basic scientific advances and potential new directions to target testosterone deficiency in SCD are being made in recent years. The objective of finding and targeting mechanism-specific endogenous sources of testosterone production appears necessary for preserving sexual function and fertility in the SCD young adult population, particularly in light of the harms of exogenous testosterone therapies.

## Author Contributions

The authors confirm contribution to the manuscript as follows: BM and AB critically reviewed the literature. BM drafted the article. BM and AB reviewed and revised the manuscript. BM and AB contributed to the article and approved the submitted version.

## Conflict of Interest

The authors declare that the research was conducted in the absence of any commercial or financial relationships that could be construed as a potential conflict of interest.

## Publisher’s Note

All claims expressed in this article are solely those of the authors and do not necessarily represent those of their affiliated organizations, or those of the publisher, the editors and the reviewers. Any product that may be evaluated in this article, or claim that may be made by its manufacturer, is not guaranteed or endorsed by the publisher.

## References

[1] Lervolino LG Baldin PE Picado SM Calil KB Viel AA Campos LA. Prevalence of Sickle Cell Disease and Sickle Cell Trait in National Neonatal Screening Studies. Rev Bras Hematol Hemoter (2011) 33:49–54. doi: 10.5581/1516-8484.20110015 23284244 PMC3521436

[2] Sundd P Gladwin MT Novelli EM. Pathophysiology of Sickle Cell Disease. Annu Rev Pathol (2019) 14:263–92. doi: 10.1146/annurev-pathmechdis-012418-012838 PMC7053558 30332562

[3] Thein MS Igbineweka NE Thein SL. Sickle Cell Disease in the Older Adult. Pathology (2017) 49:1–9. doi: 10.1016/j.pathol.2016.10.002 27914684 PMC10757825

[4] Taddesse A Woldie IL Khana P Swerdlow PS Chu JW Abrams J. Hypogonadism in Patients With Sickle Cell Disease: Central or Peripheral? Acta Haematol (2012) 128:65–8. doi: 10.1159/000337344 PMC3864664 22678347

[5] Araujo AB O'Donnell AB Brambilla DJ Simpson WB Longcope C Matsumoto AM. Prevalence and Incidence of Androgen Deficiency in Middle-Aged and Older Men: Estimates From the Massachusetts Male Aging Study. J Clin Endocrinol Metab (2004) 89:5920–26. doi: 10.1210/jc.2003-031719 15579737

[6] Abbasi AA Prasad AS Ortega J Congco E Oberleas D. Gonadal Function Abnormalities in Sickle Cell Anemia. Stud Ault Male Patients Ann Intern Med (1976) 85:601–5. doi: 10.7326/0003-4819-85-5-601 984611

[7] Abudu EK Akanmu SA Soriyan OO Akinbami AA Adediran A Adeyemo TA. Serum Testosterone Levels of HbSS (Sickle Cell Disease) Male Subjects in Lagos, Nigeria. BMC Res Notes (2011) 4:298. doi: 10.1186/1756-0500-4-298 21849076 PMC3170610

[8] Mack AK Kato GJ. Sickle Cell Disease and Nitric Oxide: A Paradigm Shift? Int J Biochem Cell Biol (2006) 38:1237–43. doi: 10.1016/j.biocel.2006.01.010 PMC2199240 16517208

[9] Nader E Conran N Romana M Connes P. Vasculopathy in Sickle Cell Disease: From Red Blood Cell Sickling to Vascular Dysfunction. Compr Physiol (2021) 11:1785–803. doi: 10.1002/cphy.c200024 33792905

[10] Kim-Shapiro DB Gladwin MT. Nitric Oxide Pathology and Therapeutics in Sickle Cell Disease. Clin Hemorheol Microcirc (2018) 68:223–37. doi: 10.3233/CH-189009 PMC5911689 29614634

[11] Osegbe DN Akinyanju OO. Testicular Dysfunction in Men With Sickle Cell Disease. Postgrad Med J (1987) 63:95–8. doi: 10.1136/pgmj.63.736.95 PMC2428240 3118348

[12] Parshad O Stevens MC Preece MA Thomas PW Serjeant GR. The Mechanism of Low Testosterone Levels in Homozygous Sickle-Cell Disease. West Indian Med J (1994) 43:12–4. 8036809

[13] Singhal A Gabay L Serjeant GR. Testosterone Deficiency and Extreme Retardation of Puberty in Homozygous Sickle-Cell Disease. West Indian Med J (1995) 44:20–3. 7793108

[14] Brachet C Heinrichs C Tenoutasse S Devalck C Azzi N Ferster A. Children With Sickle Cell Disease: Growth and Gonadal Function After Hematopoietic Stem Cell Transplantation. J Pediatr Hematol Oncol (2007) 29:445–0. doi: 10.1097/MPH.0b013e31806451ac 17609621

[15] Claudino MA Fertrin KY. Sickling Cells, Cyclic Nucleotides, and Protein Kinases: The Pathophysiology of Urogenital Disorders in Sickle Cell Anemia. Anemia (2012) 2012):723520. doi: 10.1155/2012/723520 22745902 PMC3382378

[16] Li M Fogarty J Whitney KD Stone P. Repeated Testicular Infarction in a Patient With Sickle Cell Disease: A Possible Mechanism for Testicular Failure. Urology (2003) 62:551. doi: 10.1016/s0090-4295(03)00482-5 12946770

[17] Holmes NM Kane CJ. Testicular Infarction Associated With Sickle Cell Disease. J Urol (1998) 160:130. doi: 10.1016/S0022-5347(01)63058-1 9628625

[18] Gofrit ON Rund D Shapiro A Pappo O Landau EH Pode D. Segmental Testicular Infarction Due to Sickle Cell Disease. J Urol (1998) 160:835–6. doi: 10.1097/00005392-199809010-00059 9720564

[19] Alsulmi HA. Testicular Infarction in a Patient With Sickle Cell Anemia: A Case Report. Int J Health Sci (Qassim) (2018) 12:100–2. PMC6124823 30202414

[20] el-Hazmi MA HM B al-Fawaz I. : Endocrine Functions in Sickle Cell Anaemia Patients. J Trop Pediatr (1992) 38:307–13. doi: 10.1093/tropej/38.6.307 1844090

[21] Dada OA Nduka EU. Endocrine Function and Hemoglobinopathies: Relation Between the Sickle Cell Gene and Circulating Plasma Levels of Testosterone, Luteinising Hormone (LH) and Follicle Stimulating Hormone (FSH) in Adult Males. Clin Chimica Acta (1980) 105:269–73. doi: 10.1016/0009-8981(80)90469-6 6772354

[22] Ribeiro APMR Silva CS Zambrano JCC Miranda JOF Molina CAF Gomes CM. Compensated Hypogonadism in Men With Sickle Cell Disease. Clin Endocrinol (Oxf) (2021) 94:968–72. doi: 10.1111/cen.14428 33501675

[23] AlDallal SM AlDallal NM. Infertility Issues in Men With Sickle Cell Disease. Int J Pregn Chi Birth (2017) 2:88–90. doi: 10.15406/ipcb.2017.02.00025

[24] Jones KM Niaz MS Brooks CM Roberson SI Aguinaga MP Hills ER. Adverse Effects of a Clinically Relevant Dose of Hydroxyurea Used for the Treatment of Sickle Cell Disease on Male Fertility Endpoints. Int J Environ Res Public Health (2009) 6:1124–44. doi: 10.3390/ijerph6031124 PMC2672375 19440437

[25] Chen H Zhou L Lin CY Beattie MC Liu J Zirkin BR. Effect of Glutathione Redox State on Leydig Cell Susceptibility to Acute Oxidative Stress. Mol Cell Endocrinol (2010) 323:147–54. doi: 10.1016/j.mce.2010.02.034 PMC2875365 20206230

[26] Leisegang K Roychoudhury S Slama P Finelli R. The Mechanisms and Management of Age-Related Oxidative Stress in Male Hypogonadism Associated With Non-Communicable Chronic Disease. Antioxidants (Basel) (2021) 10:1834. doi: 10.3390/antiox10111834 34829704 PMC8615233

[27] Roychoudhury S Chakraborty S Choudhury AP Das A Jha NK Slama P. Environmental Factors-Induced Oxidative Stress: Hormonal and Molecular Pathway Disruptions in Hypogonadism and Erectile Dysfunction. Antioxidants (Basel) (2021) 10:837. doi: 10.3390/antiox10060837 34073826 PMC8225220

[28] Vona R Sposi NM Mattia L Gambardella L Straface E Pietraforte DL. Sickle Cell Disease: Role of Oxidative Stress and Antioxidant Therapy. Antioxidants (Basel) (2021) 10:296. doi: 10.3390/antiox10020296 33669171 PMC7919654

[29] Wood KC Granger DN. Sickle Cell Disease: Role of Reactive Oxygen and Nitrogen Metabolites. Clin Exp Pharmacol Physiol (2007) 34:926–32. doi: 10.1111/j.1440-1681.2007.04639.x 17645642

[30] Hoppe CC. Inflammatory Mediators of Endothelial Injury in Sickle Cell Disease. Hematol Oncol Clin North Am (2014) 28:265–86. doi: 10.1016/j.hoc.2013.11.006 24589266

[31] Musicki B Zhang Y Chen H Brown TR Zirkin BR Burnett AL. Mechanism of Testosterone Deficiency in the Transgenic Sickle Cell Mouse. PloS One (2015) 10:e0128694. doi: 10.1371/journal.pone.0128694 26023917 PMC4449127

[32] Dandona P Rosenberg MT. A Practical Guide to Male Hypogonadism in the Primary Care Setting. Int J Clin Pract (2010) 64:682–96. doi: 10.1111/j.1742-1241.2010.02355.x PMC2948422 20518947

[33] Garadah TS Hassan AB Jaradat AA Diab DE Kalafalla HO Kalifa AK. Predictors of Abnormal Bone Mass Density in Adult Patients With Homozygous Sickle-Cell Disease. Clin Med Insights Endocrinol Diabetes (2015) 7(8):35–40. doi: 10.4137/CMED.S24501 PMC4426937 25987854

[34] Ballas SK Kesen MR Goldberg MF Lutty GA Dampier C Osunkwo I. Beyond the Definitions of the Phenotypic Complications of Sickle Cell Disease: An Update on Management. ScientificWorldJournal (2012) 2012:949535. doi: 10.1100/2012/949535 22924029 PMC3415156

[35] Amr MA Amin TT Al-Omair OA. Health Related Quality of Life Among Adolescents With Sickle Cell Disease in Saudi Arabia. Pan Afr Med J (2011) 8:10. doi: 10.4314/pamj.v8i1.71057 22121419 PMC3201577

[36] Longoria JN Pugh NL Gordeuk V Hsu LL Treadwell M King AA. Patient-Reported Neurocognitive Symptoms Influence Instrumental Activities of Daily Living in Sickle Cell Disease. Am J Hematol (2021) 96:1396–406. doi: 10.1002/ajh.26315 PMC8855994 34350622

[37] Huang AW Muneyyirci-Delale O. Reproductive Endocrine Issues in Men With Sickle Cell Anemia. Andrology (2017) 5:679–90. doi: 10.1111/andr.12370 28662541

[38] Osegbe DN Akinyanju O Amaku EO. Fertility in Males With Sickle Cell Disease. Lancet (1981) 2:275–6. doi: 10.1016/s0140-6736(81)90525-0 6114323

[39] Modebe O Ezeh UO. Effect of Age on Testicular Function in Adult Males With Sickle Cell Anemia. Fertil Steril (1995) 63:907–12. doi: 10.1016/s0015-0282(16)57500-1 7890081

[40] Berthaut I Guignedoux G Kirsch-Noir F de Larouziere V Ravel C Bachir D. Influence of Sickle Cell Disease and Treatment With Hydroxyurea on Sperm Parameters and Fertility of Human Males. Haematologica (2008) 93:988–93. doi: 10.3324/haematol.11515 18508803

[41] Montague DK Jarow J Broderick GA Dmochowski RR Heaton JP Lue TF. Members of the Erectile Dysfunction Guideline Update Panel; American Urological Association. American Urological Association Guideline on the Management of Priapism. J Urol (2003) 170:1318–24. doi: 10.1097/01.ju.0000087608.07371.ca 14501756

[42] Ericson C Baird B Broderick GA. Management of Priapism: 2021 Update. Urol Clin North Am (2021) 48:565–76. doi: 10.1016/j.ucl.2021.07.003 34602176

[43] Arduini GAO Trovó de Marqui AB. Prevalence and Characteristics of Priapism in Sickle Cell Disease. Hemoglobin (2018) 42:73–7. doi: 10.1080/03630269.2018.1452760 29745276

[44] Burnett AL Bivalacqua TJ. Priapism: Current Principles and Practice. Urol Clin North Am (2007) 34:631–42. doi: 10.1016/j.ucl.2007.08.006 17983902

[45] Joice GA Liu JL Burnett AL. Medical Treatment of Recurrent Ischaemic Priapism: A Review of Current Molecular Therapeutics and a New Clinical Management Paradigm. BJU Int (2021) 127:498–506. doi: 10.1111/bju.15370 33606327

[46] Addis G Spector R Shaw E Musumadi L Dhanda C. The Physical, Social and Psychological Impact of Priapism on Adult Males With Sickle Cell Disorder. Chronic Illn (2007) 3:145–54. doi: 10.1177/1742395307081505 18083669

[47] Anele UA Burnett AL. Erectile Dysfunction After Sickle Cell Disease-Associated Recurrent Ischemic Priapism: Profile and Risk Factors. J Sex Med (2015) 12:713–9. doi: 10.1111/jsm.12816 PMC4437763 25572153

[48] Burnett AL Kan-Dobrosky N Miller MG. Testosterone Replacement With 1% Testosterone Gel and Priapism: No Definite Risk Relationship. J Sex Med (2013) 10:1151–61. doi: 10.1111/jsm.12059 23347341

[49] Morrison BF Reid M Madden W Burnett AL. Testosterone Replacement Therapy Does Not Promote Priapism in Hypogonadal Men With Sickle Cell Disease: 12-Month Safety Report. Andrology (2013) 1:576–82. doi: 10.1111/j.2047-2927.2013.00084.x 23606509

[50] Podlasek CA Mulhall J Davies K Wingard CJ Hannan JL Bivalacqua TJ. Translational Perspective on the Role of Testosterone in Sexual Function and Dysfunction. J Sex Med (2016) 13:1183–98. doi: 10.1016/j.jsxm.2016.06.004 PMC5333763 27436075

[51] Musicki B Karakus S Akakpo W Silva FH Liu J Chen H. Testosterone Replacement in Transgenic Sickle Cell Mice Controls Priapic Activity and Upregulates PDE5 Expression and eNOS Activity in the Penis. Andrology (2018) 6:184–91. doi: 10.1111/andr.12442 PMC5745275 29145710

[52] Champion HC Bivalacqua TJ Takimoto E Kass DA Burnett AL. Phosphodiesterase-5a Dysregulation in Penile Erectile Tissue is a Mechanism of Priapism. Proc Natl Acad Sci USA (2005) 102:1661–6. doi: 10.1073/pnas.0407183102 PMC547836 15668387

[53] Bivalacqua TJ Musicki B Hsu LL Berkowitz DE Champion HC Burnett AL. Sildenafil Citrate-Restored eNOS and PDE5 Regulation in Sickle Cell Mouse Penis Prevents Priapism *via* Control of Oxidative/Nitrosative Stress. PloS One (2013) 8:e68028. doi: 10.1371/journal.pone.0068028 23844149 PMC3699477

[54] Silva FH Karakus S Musicki B Matsui H Bivalacqua TJ Dos Santos JL. Beneficial Effect of the Nitric Oxide Donor Compound 3-(1,3-Dioxoisoindolin-2-Yl)Benzyl Nitrate on Dysregulated Phosphodiesterase 5, NADPH Oxidase, and Nitrosative Stress in the Sickle Cell Mouse Penis: Implication for Priapism Treatment. J Pharmacol Exp Ther (2016) 359:230–7. doi: 10.1124/jpet.116.235473 PMC5074485 27540002

[55] Sopko NA Matsui H Hannan JL Berkowitz D Champion HC Hsu LL. Subacute Hemolysis in Sickle Cell Mice Causes Priapism Secondary to NO Imbalance and PDE5 Dysregulation. J Sex Med (2015) 12:1878–85. doi: 10.1111/jsm.12976 PMC4600325 26346631

[56] Lagoda G Sezen SF Cabrini MR Musicki B Burnett AL. Molecular Analysis of Erection Regulatory Factors in Sickle Cell Disease Associated Priapism in the Human Penis. J Urol (2013) 189:762–8. doi: 10.1016/j.juro.2012.08.198 PMC4478587 22982429

[57] Lin CS Chow S Lau A Tu R Lue TF. Identification and Regulation of Human PDE5A Gene Promoter. Biochem Biophys Res Commun (2001) 280:684–92. doi: 10.1006/bbrc.2000.4220 11162575

[58] Corbin JD Turko IV Beasley A Francis SH. Phosphorylation of Phosphodiesterase-5 by Cylic Nucleotide-Dependent Protein Kinase Alters its Catalytic and Allosteric cGMP-Binding Activities. Eur J Biochem (2000) 267:2760–7. doi: 10.1046/j.1432-1327.2000.01297.x 10785399

[59] Goglia L Tosi V Sanchez AM Flamini MI Fu X-D Zullino S. Endothelial Regulation of eNOS, PAI-1 and T-PA by Testosterone and Dihydrotestosterone *In Vitro* and In Vivo. Mol Hum Reprod (2010) 16:761–9. doi: 10.1093/molehr/gaq049 20547636

[60] Skogastierna C Hotzen M Rane A Ekström L. A Supraphysiological Dose of Testosterone Induces Nitric Oxide Production and Oxidative Stress. Eur J Prev Cardiol (2014) 21:1049–54. doi: 10.1177/2047487313481755 23471592

[61] Alves JV da Costa RM Pereira CA Fedoce AG Silva CAA Carneiro FS. Supraphysiological Levels of Testosterone Induce Vascular Dysfunction *via* Activation of the NLRP3 Inflammasome. Front Immunol (2020) 11:1647. doi: 10.3389/fimmu.2020.01647 32849566 PMC7411079

[62] Donaldson JF Davis N Davies JH Rees RW Steinbrecher HA. Priapism in Teenage Boys Following Depot Testosterone. J Pediatr Endocrinol Metab (2012) 25:1173–6. doi: 10.1515/jpem-2012-0270 23329767

[63] Ichioka K Utsunomiya N Kohei N Ueda N Inoue K Terai A. Testosterone-Induced Priapism in Klinefelter Syndrome. Urology (2006) 67:622.e17–8. doi: 10.1016/j.urology.2005.09.041 16504257

[64] Shergill IS Pranesh N Hamid R Arya M Anjum I. Testosterone Induced Priapism in Kallmann's Syndrome. J Urol (2003) 169:1089. doi: 10.1097/01.ju.0000049199.37765.c9 12576857

[65] Le B Chen H Zirkin B Burnett AL. New Targets for Increasing Endogenous Testosterone Production: Clinical Implications and Review of the Literature. Andrology (2014) 2:484–90. doi: 10.1111/j.2047-2927.2014.00225.x 24817562

[66] Khodamoradi K Khosravizadeh Z Parmar M Kuchakulla M Ramasamy R Arora H. Exogenous Testosterone Replacement Therapy Versus Raising Endogenous Testosterone Levels: Current and Future Prospects. F S Rev (2021) 2:32–42. doi: 10.1016/j.xfnr.2020.11.001 33615283 PMC7894643

[67] Bhasin S Brito JP Cunningham GR Hayes FJ Hodis HN Matsumoto AM. Testosterone Therapy in Men With Hypogonadism: An Endocrine Society Clinical Practice Guideline. J Clin Endocrinol Metab (2018) 103:1715–44. doi: 10.1210/jc.2018-00229 29562364

[68] Corona G Goulis DG Huhtaniemi I Zitzmann M Toppari J Forti G. European Academy of Andrology (EAA) Guidelines on Investigation, Treatment and Monitoring of Functional Hypogonadism in Males: Endorsing Organization: European Society of Endocrinology. Andrology (2020) 8:970–87. doi: 10.1111/andr.12770 32026626

[69] Barbonetti A D'Andrea S Francavilla S. : Testosterone Replacement Therapy. Andrology (2020) 8:1551–66. doi: 10.1111/andr.12774 32068334

[70] McCullough A. Alternatives to Testosterone Replacement: Testosterone Restoration. Asian J Androl (2015) 17:201–5. doi: 10.4103/1008-682X.143736 PMC4650464 25578932

[71] Trussell JC. Male Reproductive Endocrinology: When to Replace Gonadotropins. Semin Reprod Med (2013) 31:237–44. doi: 10.1055/s-0033-1345270 23775378

[72] Aghazadeh Y Zirkin BR Papadopoulos V. Pharmacological Regulation of the Cholesterol Transport Machinery in Steroidogenic Cells of the Testis. Vitam Horm (2015) 98:189–227. doi: 10.1016/bs.vh.2014.12.006 25817870

[73] Papadopoulos V Aghazadeh Y Fan J Campioli E Zirkin B Midzak A. Translocator Protein-Mediated Pharmacology of Cholesterol Transport and Steroidogenesis. Mol Cell Endocrinol (2015) 408:90–8. doi: 10.1016/j.mce.2015.03.014 PMC4417383 25818881

[74] Musicki B Karakus S La Favor JD Chen H Silva FH Sturny M. TSPO Ligand FGIN-1-27 Controls Priapism in Sickle Cell Mice *via* Endogenous Testosterone Production. J Cell Physiol (2021) 236:3073–82. doi: 10.1002/jcp.30075 PMC7882022 32974910

[75] Papadopoulos V Mukhin AG Costa E Krueger KE. The Peripheral-Type Benzodiazepine Receptor is Functionally Linked to Leydig Cell Steroidogenesis. J Biol Chem (1990) 265:3772–9. doi: 10.1016/S0021-9258(19)39661-9 2154488

[76] Culty M Luo L Yao ZX Chen H Papadopoulos V Zirkin BR. Cholesterol Transport, Peripheral Benzodiazepine Receptor, and Steroidogenesis in Aging Leydig Cells. J Androl (2002) 23:439–47. doi: 10.1002/j.1939-4640.2002.tb02251.x 12002446

[77] Krueger KE Papadopoulos V. Peripheral-Type Benzodiazepine Receptors Mediate Translocation of Cholesterol From Outer to Inner Mitochondrial Membranes in Adrenocortical Cells. J Biol Chem (1990) 265:15015–22. doi: 10.1016/S0021-9258(18)77217-7 2168398

[78] Chung JY Chen H Midzak A Burnett AL Papadopoulos V Zirkin BR. Drug Ligand-Induced Activation of Translocator Protein (TSPO) Stimulates Steroid Production by Aged Brown Norway Rat Leydig Cells. Endocrinology (2013) 154:2156–65. doi: 10.1210/en.2012-2226 PMC3740486 23525219

[79] Chung JY Brown S Chen H Liu J Papadopoulos V Zirkin B. Effects of Pharmacologically Induced Leydig Cell Testosterone Production on Intratesticular Testosterone and Spermatogenesis Biol Reprod (2020)102 489–98doi: 10.1093/biolre/ioz174 PMC7443349 31504200

[80] Tu LN Morohaku K Manna PR Pelton SH Butler WR Stocco DM. Peripheral Benzodiazepine Receptor/Translocator Protein Global Knock-Out Mice are Viable With No Effects on Steroid Hormone Biosynthesis. J Biol Chem (2014) 289:27444–54. doi: 10.1074/jbc.M114.578286 PMC4183784 24936060

[81] Morohaku K Pelton SH Daugherty DJ Butler WR Deng W Selvaraj V. Translocator Protein/Peripheral Benzodiazepine Receptor is Not Required for Steroid Hormone Biosynthesis. Endocrinology (2014) 155:89–97. doi: 10.1210/en.2013-1556 24174323 PMC3868810

[82] Selemidis S Dusting GJ Peshavariya H Kemp-Harper BK Drummond GR. Nitric Oxide Suppresses NADPH Oxidase-Dependent Superoxide Production by S-Nitrosylation in Human Endothelial Cells. Cardiovasc Res (2007) 75:349–58. doi: 10.1016/j.cardiores.2007.03.030 17568572

[83] Duerrschmidt N Stielow C Muller G Pagano PJ Morawietz H. NO-Mediated Regulation of NAD(P)H Oxidase by Laminar Shear Stress in Human Endothelial Cells. J Physiol (2006) 576:557–67. doi: 10.1113/jphysiol.2006.111070 PMC1890367 16873416

[84] Harrison CB Drummond GR Sobey CG Selemidis S. Evidence That Nitric Oxide Inhibits Vascular Inflammation and Superoxide Production *via* a P47phox-Dependent Mechanism in Mice. Clin Exp Pharmacol Physiol (2010) 37:429–34. doi: 10.1111/j.1440-1681.2009.05317.x 19843095

[85] Hamed MA Akhigbe TM Akhigbe RE Aremu AO Oyedokun PA Gbadamosi JA. Glutamine Restores Testicular Glutathione-Dependent Antioxidant Defense and Upregulates NO/cGMP Signaling in Sleep Deprivation-Induced Reproductive Dysfunction in Rats. BioMed Pharmacother (2022) 148:112765. doi: 10.1016/j.biopha.2022.112765 35247715

[86] Power-Hays A McGann PT. When Actions Speak Louder Than Words - Racism and Sickle Cell Disease. N Engl J Med (2020) 383:1902–3. doi: 10.1056/NEJMp2022125 32871062

[87] Lee L Smith-Whitley K Banks S Puckrein G. Reducing Health Care Disparities in Sickle Cell Disease: A Review. Public Health Rep (2019) 134:599–607. doi: 10.1177/0033354919881438 31600481 PMC6832089

